# Posturography assessment of balance and mobility in chronic stroke: posturography variables associated with functional performance

**DOI:** 10.3389/fmed.2026.1850883

**Published:** 2026-07-23

**Authors:** Mohammad A. ALMohiza, Ravi Shankar Reddy

**Affiliations:** 1 Department of Rehabilitation Sciences, College of Applied Medical Sciences, King Saud University, Riyadh, Saudi Arabia; 2 Program of Physical Therapy, Department of Medical Rehabilitation Sciences, College of Applied Medical Sciences, King Khalid University, Abha, Saudi Arabia

**Keywords:** activities of daily living, gait, postural balance, posturography, rehabilitation, stroke

## Abstract

**Introduction:**

Postural instability is a major contributor to impaired mobility and increased fall risk in individuals with chronic stroke. While clinical balance assessments are widely used, they may not fully capture underlying postural control deficits. This study aimed to examine the relationship between posturographic measures and clinical balance and mobility outcomes, and to determine the independent associations between posturographic variables and functional performance in chronic stroke.

**Methods:**

In this cross-sectional study, 56 ambulatory individuals with chronic stroke underwent static posturographic assessment under eyes-open, eyes-closed, and foam-surface conditions. Key parameters included the center-of-pressure path length, sway velocity, sway area, and mediolateral displacement. Clinical outcomes were assessed using the Berg Balance Scale (BBS), Timed Up and Go (TUG), 10-Meter Walk Test, Five Times Sit-to-Stand (FTSTS), Functional Reach Test (FRT), and Barthel Index. Pearson correlation and multiple linear regression analyses were performed to examine associations and identify variables independently associated with functional performance.

**Results:**

Posturographic measures significantly deteriorated under sensory-challenged conditions (*p* < 0.001). Strong correlations were observed between sway velocity (eyes closed) and BBS (*r* = −0.60, *p* < 0.001) and TUG (*r* = 0.58, *p* < 0.001). Fallers demonstrated significantly poorer balance and mobility outcomes compared to non-fallers (*p* < 0.001). Regression analysis identified sway velocity (β = 0.38 for TUG; β = −0.40 for BBS) and mediolateral sway (β = 0.34; β = −0.36) as significant, independently associated variables of functional performance.

**Discussion:**

Posturographic measures are strongly associated with clinical balance and mobility outcomes and serve as significant independent variables of functional performance in individuals with chronic stroke. These findings highlight the clinical utility of posturography as an objective tool for functional assessment and support its integration into rehabilitation planning to improve balance and mobility outcomes.

## Introduction

Postural control is a fundamental component of functional mobility and independence, requiring the integration of sensory, motor, and cognitive systems to maintain stability during both static and dynamic activities (1). In individuals with chronic stroke, impairments in postural stability are highly prevalent due to disruptions in sensorimotor integration, muscle coordination, and anticipatory postural adjustments (2). These deficits contribute significantly to reduced balance, impaired gait, and an increased risk of falls, which remain a major concern in long-term stroke rehabilitation (3). Clinical assessment of balance commonly relies on functional scales; however, such measures may not fully capture subtle impairments in postural control mechanisms, particularly under varying sensory conditions (1, 4).

Previous research has highlighted the value of posturography as an objective method to quantify postural sway and balance control (5, 6). Studies have demonstrated that increased center-of-pressure displacement and sway velocity are associated with poorer balance performance and functional limitations in stroke populations (7, 8). Awosika et al. (9) reported that altered sensory integration significantly affects postural stability, particularly when visual input is reduced. Similarly, ALMohiza et al. (4) identified postural asymmetry and impaired weight distribution as key contributors to functional mobility deficits, while Sidaway et al. (10) emphasized the role of mediolateral instability being associated with fall risk and mobility impairment. Additionally, evidence suggests that posturographic measures obtained under eyes-closed conditions may provide greater sensitivity for detecting balance impairments than standard clinical assessments (11).

Despite these advances, important gaps remain in understanding how objective posturographic parameters relate to clinically meaningful outcomes across multiple domains of function in individuals with chronic stroke. Many studies have examined either laboratory-based balance measures or clinical outcomes in isolation, limiting the ability to establish comprehensive relationships between postural control and functional performance (1, 12, 13). Furthermore, the clinical relevance of specific posturographic variables, particularly those reflecting directional instability, for mobility and balance outcomes remains insufficiently clarified in clinically relevant populations (14). Addressing this gap is essential for improving assessment strategies and guiding targeted rehabilitation interventions. In contrast to previous studies that primarily established general associations between posturography and balance impairment after stroke, the present study specifically examined whether distinct posturography variables provide differential clinical value for functional assessment. Particular emphasis was placed on mediolateral sway and sway velocity under sensory-challenged conditions, including eyes-closed testing, as these parameters may more sensitively reflect deficits in sensorimotor integration and dynamic stability relevant to mobility performance and fall risk. In addition, this study evaluated the associations of these variables across multiple functional domains, including balance, gait performance, sit-to-stand ability, and activities of daily living, thereby providing a more comprehensive characterization of posturography’s clinical utility in chronic stroke rehabilitation.

The present study aimed to investigate the relationships between posturographic measures and clinical balance and mobility outcomes, and to determine the extent to which posturographic variables are independently associated with functional performance in individuals with chronic stroke. In addition, exploratory subgroup analyses were performed to examine differences in posturographic and clinical measures between participants classified as fallers and non-fallers. However, these exploratory subgroup analyses primarily focused on differences in clinical and functional outcome measures between groups rather than direct comparisons of individual posturographic parameters. Comparative analyses of posturographic variables between fallers and non-fallers may provide additional clinically relevant insight into fall-related balance impairments and should be considered in future investigations. It was hypothesized that greater postural instability, particularly under sensory-challenged conditions, would be significantly associated with poorer balance and mobility outcomes, and that key posturographic parameters, including sway velocity and mediolateral displacement, would emerge as significant, independently associated variables of functional performance.

## Material and methods

### Study design, ethics, and settings

This cross-sectional study was conducted between January 2025 and December 2025 at the Neurorehabilitation Clinic, Department of Rehabilitation Sciences, King Khalid University, Kingdom of Saudi Arabia. Ethical approval was obtained from the Institutional Review Board of King Khalid University (KKU-166-2025-31), and written informed consent was obtained from all participants prior to enrollment. The study was carried out in accordance with the ethical principles outlined in the Declaration of Helsinki.

### Sample size calculation

The sample size was calculated using G*Power based on a two-tailed correlation analysis examining the relationship between posturography and clinical outcomes in chronic stroke (11). Assuming a moderate effect size (*r* = 0.37), α = 0.05, and power = 0.80, the required sample size was 53 participants. This was increased to 56 to account for potential data loss and ensure sufficient power for the planned analyses. The assumed effect size was based on previously reported correlations between posturographic sway parameters, particularly sway velocity measures, and clinical balance and mobility outcomes in individuals with chronic stroke (15), and was selected to reflect the primary planned correlation analyses rather than a single predefined pair of variables.

### Participants

Participants were recruited from the outpatient Neurorehabilitation Clinic through consecutive sampling of individuals referred for balance and mobility rehabilitation. Additional participants were identified through clinician referrals within the department. Eligibility was determined based on a confirmed diagnosis of first-ever unilateral ischemic or hemorrhagic stroke, established by a neurologist using clinical examination and neuroimaging reports in accordance with standard stroke diagnostic criteria (16). All potential participants underwent an initial screening process that included medical record review and a brief clinical assessment to verify eligibility. Individuals were included if they were in the chronic stage of stroke (≥ 6 months post-onset), aged 40 years or older, able to stand independently for at least 30 s, and capable of walking at least 10 meters with or without an assistive device. Participants were excluded if they had cerebellar involvement, other neurological conditions affecting balance, severe musculoskeletal impairments that limited standing or walking, significant visual or vestibular disorders, or cognitive or communication impairments that interfered with understanding of instructions. Baseline assessments were conducted prior to enrollment to confirm functional ability and ensure safe participation in posturographic and clinical testing procedures. Additional variables such as cognitive status, lesion location, stroke severity, and psychological factors, including fear of falling, were not systematically evaluated within the present study protocol. Consequently, these factors were not incorporated into the regression analyses, although they may contribute to variability in postural control and functional performance among individuals with chronic stroke.

### Posturographic parameters

Posturographic variables were assessed using a calibrated force platform system to quantify static postural control under different sensory conditions (17, 18). The force platform system (Iso-free, Technobody, Italy) was calibrated prior to testing according to the manufacturer’s guidelines, and data were collected using proprietary software with a sampling frequency of 100 Hz (4). Participants were instructed to stand barefoot on the platform with feet positioned at a standardized width (approximately hip-width apart), and arms relaxed alongside the body. Foot position was standardized with heels placed 10 cm apart and feet oriented at approximately 30° of external rotation. A rest interval of at least 60 s was provided between trials to minimize fatigue. All assessments were conducted in a quiet, well-lit environment at a consistent time of day to reduce variability. Participants were assessed while on stable medication and were instructed to avoid strenuous physical activity prior to testing. Three conditions were assessed: eyes open on a firm surface, eyes closed on a firm surface, and eyes open on a medium-density foam surface placed over the platform to alter somatosensory input. The foam surface consisted of a medium-density compliant balance pad (approximately 50 × 41 × 6 cm) made of closed-cell foam, comparable to commercially available rehabilitation balance pads used for postural stability assessment and sensory perturbation protocols. A fourth condition involving eyes closed on a foam surface was not included because simultaneous visual deprivation and unstable surface support substantially increase postural challenge and fall risk in individuals with chronic stroke. The selected three-condition protocol was adopted to ensure participant safety while still allowing assessment of sensory contributions to postural control. Each trial lasted 30 s, and three trials per condition were recorded, with the mean value used for analysis.

The primary posturographic variables included center of pressure (COP) path length (mm), mean sway velocity (mm/s), sway area (mm^2^), anteroposterior (AP) displacement (mm), mediolateral (ML) displacement (mm), root mean square (RMS) sway in the AP and ML directions (mm), 95% confidence ellipse area (mm^2^), and the Romberg ratio, calculated as the ratio of eyes-closed to eyes-open sway measures (19). These variables were derived from COP time-series data sampled at 100 Hz and processed with the manufacturer’s software (19). Higher values indicated greater postural instability (19).

### Clinical balance outcome (Berg Balance Scale)

Balance performance was assessed using the Berg Balance Scale (BBS), a 14-item performance-based measure evaluating static and dynamic balance tasks commonly encountered in daily activities (20). Each item was scored on a 5-point ordinal scale (0–4), with a total score ranging from 0 to 56, where higher scores indicate better balance (20). The assessment was conducted by a trained physiotherapist in accordance with standardized administration guidelines, including demonstrations and verbal instructions for each task. Participants were allowed one practice trial, where appropriate, to ensure comprehension. The BBS has been extensively validated in stroke populations, demonstrating high inter-rater reliability and sensitivity to balance impairments (21, 22). Scores below 45 were considered indicative of increased fall risk, in line with established clinical thresholds (21).

### Functional mobility outcome (Timed Up and Go Test)

Functional mobility was evaluated using the Timed Up and Go (TUG) test, which measures the time required for a participant to stand up from a standard chair (seat height ∼45 cm), walk 3 meters at a comfortable pace, turn, return, and sit down (23). Time was recorded in seconds using a stopwatch, with shorter times indicating better mobility. Participants performed one familiarization trial followed by two recorded trials, and the mean value was used for analysis. Standardized instructions were provided, and assistive devices were permitted if regularly used. The TUG is a reliable and valid measure of functional mobility and fall risk in stroke populations (24, 25), with values greater than 13.5 s commonly associated with increased fall risk (24).

### Gait speed (10-Meter walk test)

Gait performance was assessed using the 10-Meter Walk Test (10MWT), conducted on a straight 14-meter walkway to allow for acceleration and deceleration phases (26). The middle 10 meters were timed to calculate steady-state walking speed (m/s) (26). Participants were instructed to walk at their comfortable speed, and two trials were performed with the average value used for analysis (26). Timing was recorded using a handheld stopwatch. The 10MWT is a widely accepted and reliable measure of gait speed in stroke rehabilitation (27), with speeds below 0.8 m/s generally indicating limited community ambulation (27).

### Functional endurance (6-Minute walk test)

Functional walking endurance was assessed using the 6-Minute Walk Test (6MWT), conducted along a 30-meter straight indoor walkway in accordance with standardized guidelines (28). Participants were instructed to walk as far as possible within 6 min at a self-selected pace, with standardized encouragement provided at regular intervals. Total distance covered (meters) was recorded (28). Rest breaks were permitted if required, but included within the total test duration. The 6MWT is a valid and reliable measure of functional endurance and community ambulation capacity in individuals with stroke (29, 30).

### Lower limb functional strength (Five Times Sit-to-Stand Test)

Lower-limb functional strength was assessed using the Five Times Sit-to-Stand Test (FTSTS), which measures the time required to complete five consecutive sit-to-stand cycles from a standardized chair without using the upper limbs (31). Participants were instructed to perform the task as quickly as possible while maintaining safety. During the test, participants were instructed to keep their arms crossed over their chests throughout task performance to minimize upper-limb assistance and ensure standardized testing conditions. Time was recorded in seconds, with longer durations indicating reduced lower limb strength and functional performance (31). One practice trial was provided, followed by one recorded trial. The FTSTS has demonstrated good reliability and validity in neurological populations and is sensitive to functional impairments in stroke (32).

### Dynamic balance (Functional Reach Test)

Dynamic balance was evaluated using the Functional Reach Test (FRT), which measures the maximum forward reach distance (cm) while maintaining a fixed base of support (33). Participants stood upright with feet shoulder-width apart and were instructed to reach forward as far as possible without stepping or losing balance. The distance was measured with a wall-mounted tape measure aligned with the acromion process. Three trials were conducted, and the maximum value was used for analysis. The FRT is a simple and reliable measure of dynamic balance and has been validated in stroke populations (34).

### Functional independence (Barthel Index)

Functional independence in activities of daily living was assessed using the Barthel Index (BI), which evaluates performance in 10 domains, including feeding, bathing, grooming, dressing, transfers, mobility, and continence (35). Scores range from 0 to 100, with higher scores indicating greater independence (35). The assessment was conducted through structured interviews and observation, following standardized scoring criteria (35).

### Demographic and clinical covariates

Demographic and clinical variables included age (years), sex (male/female), body mass index (BMI, kg/m^2^), stroke type (ischemic or hemorrhagic), affected side (left/right), time since stroke (months), and use of assistive devices (yes/no). These variables were obtained from medical records and participant interviews. Fall history was defined as the occurrence of at least one unexpected event in which the participant came to rest on the ground, floor, or lower level within the previous 6 months. The assessment recorded at least one fall during the specified period and was used to classify participants as fallers or non-fallers; the exact number of falls was not recorded. These covariates were included to describe the sample and to control for potential confounding effects in regression analyses.

### Statistical analysis

All statistical analyses were performed using IBM SPSS Statistics for Windows, version 24.0 (IBM Corp., Armonk, NY, USA). To improve interpretability and avoid overrepresenting measurement precision, descriptive and outcome variables across all tables were reported as rounded values consistent with the data’s variability and scale. Data were initially screened for completeness and normality using the Shapiro–Wilk test and visual inspection of histograms, confirming that all continuous variables were normally distributed. Descriptive statistics were calculated as mean ± standard deviation for continuous variables and frequencies with percentages for categorical variables. For Objective 1, differences in posturographic parameters across sensory conditions (eyes open, eyes closed, and foam surface) were analyzed using repeated-measures analysis of variance (ANOVA) with Greenhouse–Geisser correction where appropriate, followed by Bonferroni-adjusted pairwise comparisons. Pearson correlation coefficients were computed to examine the relationships between posturographic variables and clinical balance, mobility, and functional outcomes. For Objective 2, multiple linear regression analyses (enter method) were performed to identify independently associated variables of functional mobility (Timed Up and Go) and balance (Berg Balance Scale), including relevant posturographic variables and clinical covariates. Assumptions of linear regression, including linearity, independence of errors, homoscedasticity, and normality of residuals, were evaluated prior to analysis. The number of independently associated variables included in each model was selected based on theoretical relevance and sample size considerations to avoid model overfitting. Multicollinearity was assessed using variance inflation factors prior to model estimation. A *p*-value of < 0.05 was considered statistically significant for all analyses. Because multiple statistical tests were performed, *p*-values were not adjusted for multiple comparisons and should be interpreted as exploratory.

## Results

Fallers demonstrated consistently poorer balance, mobility, and functional performance compared with non-fallers across multiple clinical outcome measures, while demographic and stroke-related characteristics were generally comparable between groups (Table 1). Lower Berg Balance Scale scores, slower gait speed, prolonged Timed Up and Go and Five Times Sit-to-Stand performance, and reduced Functional Reach Test values were observed among fallers, indicating clinically meaningful impairments in balance control, mobility, and lower-limb functional performance. Fallers also showed lower Barthel Index scores, suggesting reduced independence in activities of daily living. In contrast, body mass index and time since stroke appeared relatively similar between groups.

**TABLE 1:** Demographic and clinical characteristics of ambulatory individuals with chronic stroke included in the cross-sectional study, overall and stratified by fall history (*n* = 56).

Variable	Total (*n* = 56)	Non-fallers (*n* = 35)	Fallers (*n* = 21)
Age, years	61.8 ± 8.8	59.9 ± 8.1	65.0 ± 9.2
Sex, male, *n* (%)	34 (60.7)	20 (57.1)	14 (66.6)
Body mass index, kg/m^2^	26.2 ± 3.6	25.7 ± 3.4	27.0 ± 3.9
Time since stroke, months	19.1 ± 8.8	17.8 ± 8.4	21.2 ± 9.3
Stroke type, ischemic, *n* (%)	46 (82.1)	29 (82.8)	17 (80.9)
Affected side, right, *n* (%)	28 (50.0)	18 (51.4)	10 (47.6)
Use of assistive device, *n* (%)	18 (32.1)	8 (22.8)	10 (47.6)
Barthel Index, points	85.4 ± 10.1	88.7 ± 8.5	79.9 ± 10.1
Berg Balance Scale, points	43.1 ± 7.1	45.8 ± 5.9	38.6 ± 6.7
Timed Up and Go, s	15.9 ± 4.5	14.3 ± 3.2	18.7 ± 4.9
10-Meter Walk Test speed, m/s	0.7 ± 0.2	0.8 ± 0.1	0.6 ± 0.1
Five Times Sit-to-Stand, s	17.2 ± 5.1	15.4 ± 4.2	20.1 ± 5.3
Functional Reach Test, cm	22.4 ± 5.8	24.6 ± 5.1	18.7 ± 4.9
Participants with ≥ 1 fall in the previous 6 months, *n* (%)	21 (37.5)	N/A	21 (100.0)

BMI, body mass index; m/s, meters per second. Values are presented descriptively as mean ± standard deviation or frequency (%).

Posturographic measures demonstrated a progressive deterioration in balance performance across increasingly challenging sensory conditions, with values consistently increasing from eyes-open to eyes-closed and foam-surface standing (Table 2). COP path length increased from 412.6 mm during eyes-open standing to 684.3 mm on the foam surface, while mean sway velocity increased from 13.8 mm/s to 22.8 mm/s, indicating substantially greater postural instability under altered sensory conditions. Similar progressive increases were observed for sway area, AP displacement, ML displacement, and RMS sway measures, with large effect sizes across all parameters (partial η^2^ = 0.76–0.80). Pairwise comparisons further demonstrated significantly greater instability during foam-surface standing compared with both eyes-open and eyes-closed conditions.

**TABLE 2:** Posturographic parameters across eyes-open, eyes-closed, and foam-surface standing conditions in ambulatory individuals with chronic stroke (*n* = 56).

Parameter	Eyes open	Eyes closed	Foam surface	*p*-value	Partial η ^2^	95% CI, mean difference (EO vs. EC)	95% CI, mean difference (EO vs. Foam)
COP path length (mm)	412.60 ± 86.40	536.80 ± 109.70	684.30 ± 131.50	< 0.001	0.79	(−152.77, −95.63)	(−300.03, −243.37)
Mean sway velocity (mm/s)	13.75 ± 2.88	17.89 ± 3.66	22.81 ± 4.38	< 0.001	0.80	(−5.03, −3.25)	(−9.95, −8.17)
Sway area (mm^2^)	238.40 ± 91.30	372.70 ± 128.60	566.90 ± 181.40	< 0.001	0.76	(−172.48, −96.12)	(−366.68, −290.32)
AP displacement (mm)	9.62 ± 2.41	12.84 ± 3.14	16.93 ± 3.88	< 0.001	0.78	(−4.01, −2.43)	(−8.10, −6.52)
ML displacement (mm)	7.81 ± 2.06	10.42 ± 2.68	14.26 ± 3.37	< 0.001	0.79	(−3.30, −1.92)	(−7.14, −5.76)
RMS AP sway (mm)	3.12 ± 0.79	4.18 ± 1.03	5.66 ± 1.27	< 0.001	0.78	(−1.31, −0.81)	(−2.79, −2.29)
RMS ML sway (mm)	2.54 ± 0.68	3.46 ± 0.87	4.87 ± 1.12	< 0.001	0.80	(−1.14, −0.70)	(−2.55, −2.11)
95% ellipse area (mm^2^)	182.50 ± 74.60	296.40 ± 109.20	458.70 ± 152.80	< 0.001	0.77	(−145.98, −81.82)	(−308.28, −244.12)

AP, anteroposterior; CI, confidence interval; COP, center of pressure; EC, eyes closed; EO, eyes open; ML, mediolateral; RMS, root mean square. Foam-surface standing was performed with eyes open on a medium-density foam pad. Larger values indicate greater postural instability for all posturographic variables. Values are presented as mean ± standard deviation. Omnibus condition effects were examined using one-way repeated-measures ANOVA with Greenhouse–Geisser correction where appropriate; pairwise comparisons were Bonferroni-adjusted.

Substantial differences in balance and mobility performance were observed between fallers and non-fallers, indicating markedly reduced functional capacity among individuals with a history of falls (Table 3). Fallers demonstrated significantly lower Berg Balance Scale scores (37.10 vs. 46.80; mean difference 9.70, *p* < 0.001) and reduced functional reach (15.40 vs. 22.60 cm; *p* < 0.001), reflecting impaired balance control. Mobility limitations were evident with longer Timed Up and Go times (23.10 vs 16.40 s; *p* < 0.001) and slower gait speed (0.56 vs 0.84 m/s; *p* < 0.001). Endurance was also reduced, with a substantially lower 6-Minute Walk Test distance (173.80 vs. 247.60 m; *p* < 0.001). Additionally, fallers showed poorer lower-limb strength (FTSTS: 27.80 vs 18.70 s; *p* < 0.001) and slightly lower independence (Barthel Index: 81.80 vs. 88.50; *p* = 0.017), highlighting clinically meaningful functional deficits.

**TABLE 3:** Clinical balance and mobility outcomes in the overall cohort and according to fall status among community-dwelling individuals with chronic stroke (*n* = 56).

Outcome measure	Overall (*n* = 56)	Non-fallers (*n* = 35)	Fallers (*n* = 21)	Mean difference (95% CI)	*p*-value
Berg Balance Scale, score	43.16 ± 7.80	46.80 ± 5.90	37.10 ± 6.80	9.70 (6.24 to 13.16)	< 0.001
Timed Up and Go, s	18.91 ± 5.64	16.40 ± 4.20	23.10 ± 5.30	−6.70 (−9.27 to −4.13)	< 0.001
10-Meter Walk Test speed, m/s	0.73 ± 0.23	0.84 ± 0.19	0.56 ± 0.17	0.28 (0.18 to 0.38)	< 0.001
6-Minute Walk Test distance, m	219.92 ± 74.22	247.60 ± 69.40	173.80 ± 58.20	73.80 (37.57 to 110.03)	< 0.001
Five Times Sit-to-Stand, s	22.11 ± 7.40	18.70 ± 5.10	27.80 ± 7.20	−9.10 (−12.40 to −5.80)	< 0.001
Functional Reach Test, cm	19.90 ± 6.12	22.60 ± 5.20	15.40 ± 4.80	7.20 (4.40 to 10.00)	< 0.001
Barthel Index, score	85.99 ± 10.31	88.50 ± 9.40	81.80 ± 10.60	6.70 (1.24 to 12.16)	0.017

Values are presented as mean ± standard deviation unless otherwise indicated. Between-group comparisons were performed using independent-samples *t*-tests. Mean difference was calculated as non-fallers minus fallers. CI, confidence interval; m, meters; m/s, meters per second; s, seconds.

**FIGURE 1:**
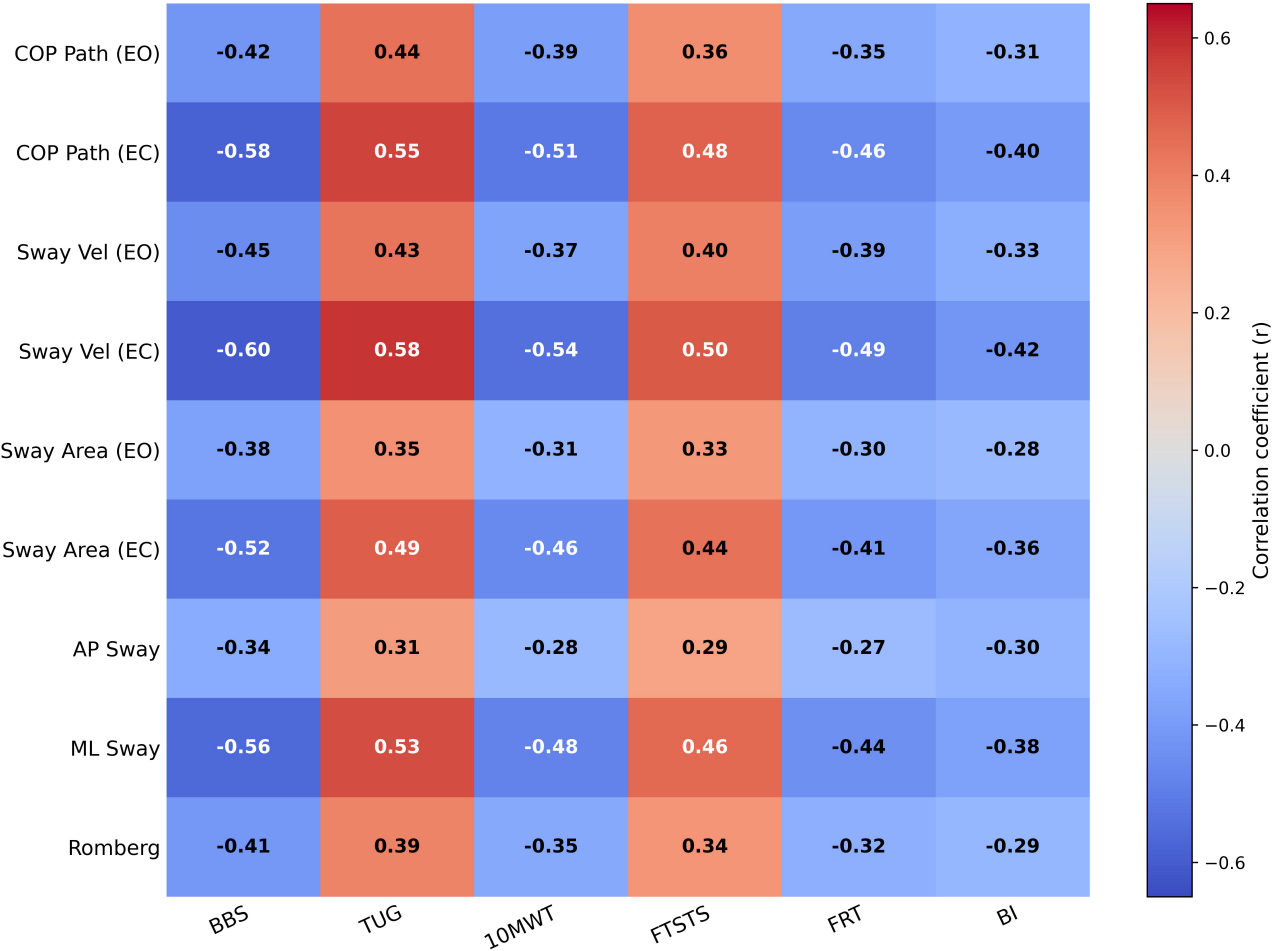
Heatmap illustrating correlations between posturographic parameters and clinical balance and mobility outcomes in individuals with chronic stroke.

Posturographic parameters demonstrated consistent and clinically meaningful correlations with balance and mobility outcomes, with stronger associations observed under eyes-closed conditions (Figure 1 and Table 4). COP path length and sway velocity (EC) showed the strongest relationships with BBS (*r* = −0.58 and -0.60, respectively; *p* < 0.001) and TUG (*r* = 0.55 and 0.58; *p* < 0.001), indicating that greater instability was associated with poorer balance and slower mobility. Similarly, ML sway exhibited robust correlations with functional measures, including BBS (*r* = −0.56) and FTSTS (*r* = 0.46), all *p* < 0.001. Negative correlations with gait speed (e.g., sway velocity EC: *r* = −0.54, *p* < 0.001) and functional reach further support reduced performance with increased instability. Overall, moderate to strong correlations (r ≈ 0.30–0.60) across variables highlight that posturographic impairments are closely linked to functional limitations in chronic stroke.

**TABLE 4:** Pearson correlation matrix between posturographic parameters under different sensory conditions and clinical balance, mobility, and functional outcomes in individuals with chronic stroke (*n* = 56).

Posturography variable	Mean ± SD	BBS	TUG, s	10MWT speed, m/s	FTSTS, s	FRT, cm	BI
		r (95% CI); p Mean ± SD: 41.82 ± 8.21	r (95% CI); p Mean ± SD: 17.34 ± 5.28	r (95% CI); p Mean ± SD: 0.74 ± 0.22	r (95% CI); p Mean ± SD: 18.91 ± 6.47	r (95% CI); p Mean ± SD: 18.26 ± 5.03	r (95% CI); p Mean ± SD: 82.45 ± 11.72
COP path length (EO), cm	52.84 ± 12.3	−0.42 (−0.61 to −0.18); 0.001	0.44 (0.20 to 0.63); < 0.001	−0.39 (−0.59 to −0.14); 0.003	0.36 (0.11 to 0.57); 0.006	−0.35 (−0.56 to −0.10); 0.008	−0.31 (−0.53 to −0.05); 0.020
COP path length (EC), cm	71.4 ± 16.5	−0.58 (−0.73 to −0.37); < 0.001	0.55 (0.34 to 0.71); < 0.001	−0.51 (−0.68 to −0.29); < 0.001	0.48 (0.25 to 0.66); < 0.001	−0.46 (−0.64 to −0.22); < 0.001	−0.40 (−0.60 to −0.15); 0.002
Sway velocity (EO), cm/s	1.76 ± 0.4	−0.45 (−0.64 to −0.21); < 0.001	0.43 (0.19 to 0.62); < 0.001	−0.37 (−0.58 to −0.12); 0.005	0.40 (0.15 to 0.60); 0.002	−0.39 (−0.59 to −0.14); 0.003	−0.33 (−0.55 to −0.07); 0.013
Sway velocity (EC), cm/s	2.38 ± 0.5	−0.60 (−0.75 to −0.40); < 0.001	0.58 (0.37 to 0.73); < 0.001	−0.54 (−0.70 to −0.32); < 0.001	0.50 (0.27 to 0.67); < 0.001	−0.49 (−0.67 to −0.26); < 0.001	−0.42 (−0.61 to −0.18); 0.001
Sway area (EO), cm^2^	3.92 ± 1.4	−0.38 (−0.58 to −0.13); 0.004	0.35 (0.10 to 0.56); 0.008	−0.31 (−0.53 to −0.05); 0.020	0.33 (0.07 to 0.55); 0.013	−0.30 (−0.52 to −0.04); 0.025	−0.28 (−0.51 to −0.02); 0.037
Sway area (EC), cm^2^	6.28 ± 2.1	−0.52 (−0.69 to −0.30); < 0.001	0.49 (0.26 to 0.67); < 0.001	−0.46 (−0.64 to −0.22); < 0.001	0.44 (0.20 to 0.63); < 0.001	−0.41 (−0.61 to −0.16); 0.002	−0.36 (−0.57 to −0.11); 0.006
AP sway, cm	1.31 ± 0.3	−0.34 (−0.55 to −0.08); 0.010	0.31 (0.05 to 0.53); 0.020	−0.28 (−0.51 to −0.02); 0.037	0.29 (0.03 to 0.51); 0.030	−0.27 (−0.50 to −0.01); 0.044	−0.30 (−0.52 to −0.04); 0.025
ML sway, cm	1.09 ± 0.3	−0.56 (−0.72 to −0.35); < 0.001	0.53 (0.31 to 0.70); < 0.001	−0.48 (−0.66 to −0.25); < 0.001	0.46 (0.22 to 0.64); < 0.001	−0.44 (−0.63 to −0.20); < 0.001	−0.38 (−0.58 to −0.13); 0.004
Romberg ratio	1.39 ± 0.2	−0.41 (−0.61 to −0.16); 0.002	0.39 (0.14 to 0.59); 0.003	−0.35 (−0.56 to −0.10); 0.008	0.34 (0.08 to 0.55); 0.010	−0.32 (−0.54 to −0.06); 0.016	−0.29 (−0.51 to −0.03); 0.030

AP, anteroposterior; BI, Barthel Index; BBS, Berg Balance Scale; CI, confidence interval; COP, center of pressure; EC, eyes closed; EO, eyes open; FRT, Functional Reach Test; FTSTS, Five Times Sit-to-Stand; ML, mediolateral; SD, standard deviation; TUG, Timed Up and Go; 10MWT, 10-Meter Walk Test. Values are presented as Pearson correlation coefficients (r) with 95% confidence intervals and two-tailed *p*-values. Negative coefficients indicate that greater postural instability was associated with poorer performance on scales in which higher scores reflect better function (BBS, FRT, BI, and 10MWT speed), whereas positive coefficients indicate greater instability was associated with longer completion times on TUG and FTSTS.

**FIGURE 2:**
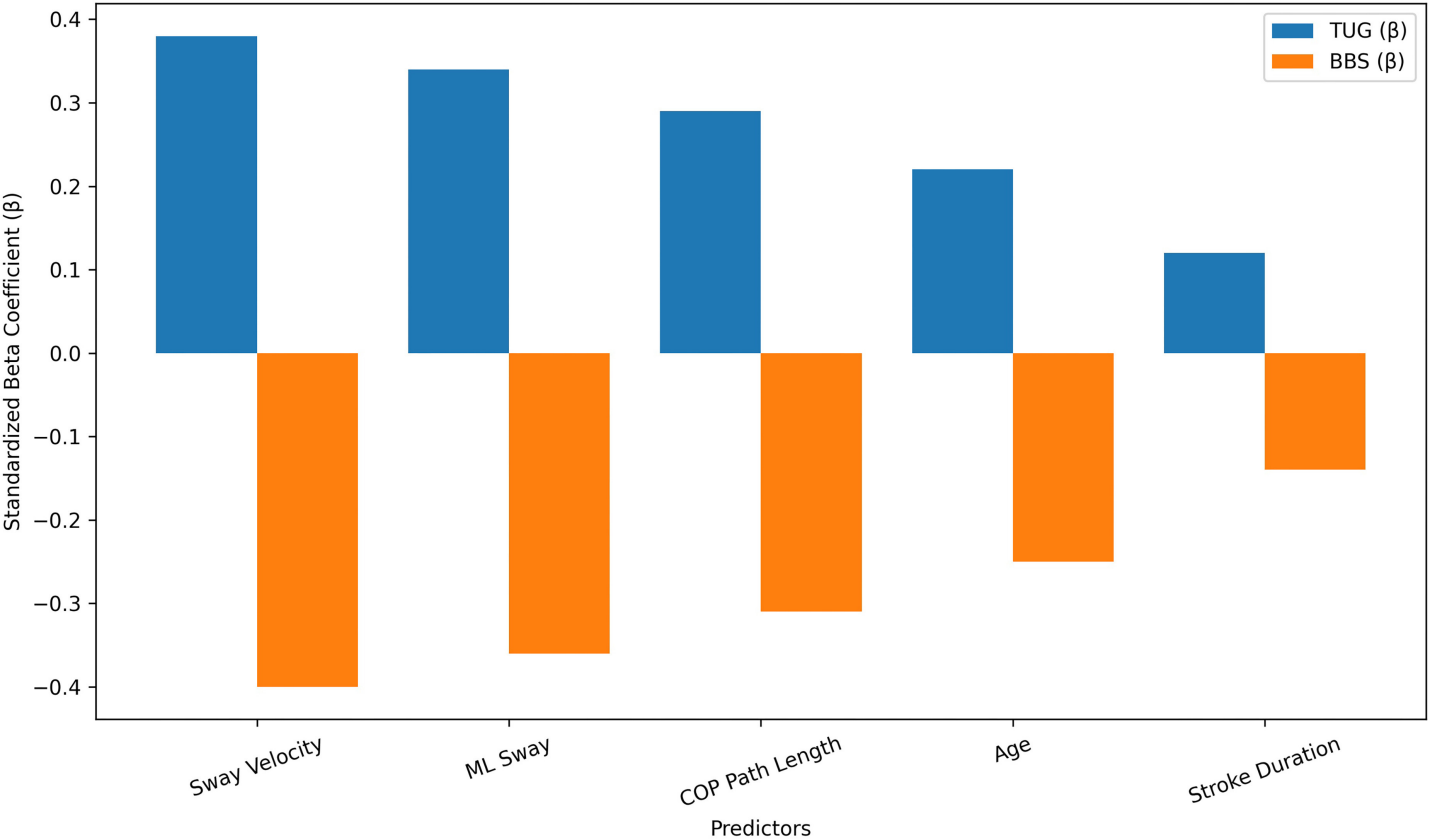
Standardized beta coefficients of posturographic and clinical predictors for functional mobility (TUG) and balance (BBS) in chronic stroke survivors.

Multiple linear regression analysis identified several posturographic variables as significantly associated with balance and mobility outcomes (Figure 2 and Table 5). For TUG, sway velocity (β = 0.38, *p* < 0.001), ML sway (β = 0.34, *p* = 0.001), and COP path length (β = 0.29, *p* = 0.003) were significant independently associated variables, indicating that greater postural instability was associated with slower mobility performance, while age showed a smaller but significant effect (β = 0.22, *p* = 0.014). For BBS, sway velocity emerged as the strongest association (β = −0.40, *p* < 0.001), followed by ML sway (β = −0.36, *p* = 0.001) and COP path length (β = −0.31, *p* = 0.006), indicating that greater instability was associated with poorer balance. Stroke duration was not a significant independent variable in either model (*p* > 0.05), suggesting that functional outcomes were more strongly influenced by current balance impairments than chronicity. For the BBS model, stroke duration showed a non-significant trend (β = −0.14, *p* = 0.098), and this finding should be interpreted with caution because it did not meet the predefined threshold for statistical significance.

**TABLE 5:** Multiple linear regression analysis examining associations with functional mobility outcomes in chronic stroke survivors (*n* = 56).

Dependent variable	Association strength	B	SE	β	95% CI	*p*-value	Tolerance	VIF
TUG (s)	Sway velocity	0.45	0.12	0.38	0.21 to 0.69	< 0.001	0.71	1.41
TUG (s)	COP path length	0.30	0.10	0.29	0.10 to 0.50	0.003	0.69	1.45
TUG (s)	ML sway	0.52	0.15	0.34	0.22 to 0.82	0.001	0.66	1.52
TUG (s)	Age	0.20	0.08	0.22	0.04 to 0.36	0.014	0.82	1.22
TUG (s)	Stroke duration	0.10	0.07	0.12	−0.04 to 0.24	0.152	0.88	1.14
BBS (score)	Sway velocity	−1.20	0.35	−0.40	−1.90 to −0.50	< 0.001	0.71	1.41
BBS (score)	COP path length	−0.80	0.28	−0.31	−1.36 to −0.24	0.006	0.69	1.45
BBS (score)	ML sway	−1.50	0.42	−0.36	−2.34 to −0.66	0.001	0.66	1.52
BBS (score)	Age	−0.60	0.20	−0.25	−1.00 to −0.20	0.004	0.82	1.22
BBS (score)	Stroke duration	−0.30	0.18	−0.14	−0.66 to 0.06	0.098	0.88	1.14

Model Statistics: TUG model: R^2^ = 0.58, Adjusted R^2^ = 0.54, *F* = 13.72, *p* < 0.001. BBS model: R^2^ = 0.62, Adjusted R^2^ = 0.58, *F* = 15.41, *p* < 0.001. Values are presented as unstandardized regression coefficients (B), standard errors (SE), standardized beta coefficients (β), 95% confidence intervals (CI), *p*-values, tolerance, and variance inflation factor (VIF). AP, anteroposterior; BBS, Berg Balance Scale; CI, confidence interval; COP, center of pressure; ML, mediolateral; SD, standard deviation; SE, standard error; TUG, Timed Up and Go; VIF, variance inflation factor.

## Discussion

This study aimed to investigate the relationships between posturographic measures of balance and clinical mobility outcomes, and to determine the extent to which postural instability is associated with functional performance in individuals with chronic stroke. The findings demonstrated that posturographic parameters were significantly associated with clinical measures of balance, mobility, and functional independence, with stronger relationships observed under more challenging sensory conditions. Individuals with a history of falls exhibited notably poorer balance, reduced mobility, and lower functional capacity compared to non-fallers. Furthermore, key posturographic variables, particularly those reflecting sway magnitude and mediolateral instability, emerged as significant, independently associated variables of both mobility performance and balance outcomes. Collectively, these results highlight the clinical relevance of objective posturographic assessment in capturing balance impairments and their impact on functional limitations in chronic stroke.

The observed associations between posturographic parameters and clinical balance and mobility outcomes can be explained by underlying deficits in sensorimotor integration and impaired postural control mechanisms following stroke (36). First, damage to cortical and subcortical structures involved in proprioceptive and vestibular processing reduces the ability to effectively integrate sensory inputs, leading to increased postural sway, particularly under sensory-challenged conditions such as eyes-closed standing (37). This is consistent with findings reported by Saeys et al. (38) who demonstrated that altered sensory weighting contributes to postural instability in stroke survivors, especially when visual input is removed. Second, impaired neuromuscular coordination and delayed postural responses reduce the efficiency of maintaining upright stability, resulting in poorer performance on functional tasks (39). Xu et al. (40) reported that postural alignment and symmetry deficits are strongly linked to balance impairments and functional mobility limitations in stroke, while Jonsdottir et al. (41) further highlighted that increased mediolateral sway is a critical determinant of functional instability and fall risk. These mechanisms may help explain the moderate-to-strong correlations observed between posturographic measures and clinical outcomes.

The observed associations between posturographic variables and functional mobility and balance outcomes can be attributed to the direct influence of deficits in postural control on task-specific motor performance and functional independence (42). One key reason is that increased sway, particularly in the mediolateral direction, reflects instability that compromises dynamic tasks such as walking, turning, and transitional movements, thereby prolonging task completion times and reducing balance scores (43). This aligns with the findings of Sidaway et al. (10), who identified mediolateral instability as a major contributor to impaired mobility and fall risk in neurological populations. Additionally, greater postural instability indicates reduced capacity for anticipatory and reactive postural adjustments, which are essential for efficient execution of functional activities (2). Roelofs et al. (44) reported that deficits in balance control significantly limit functional recovery and independence after stroke, while Pellicciari et al. (45) demonstrated that impairments in trunk and postural control are strong, independently associated variables of functional outcomes in stroke rehabilitation. Together, these findings support the observed associations between objective posturographic measures and mobility and balance performance, whereas chronicity alone has a comparatively limited influence on functional outcomes (46).

### Clinical significance

The findings of this study have important clinical implications for neurological physical therapy, particularly in the assessment and management of balance and mobility impairments in individuals with chronic stroke. The demonstrated associations between posturographic parameters and functional performance highlight the value of objective balance assessment in identifying subtle postural control deficits that may not be fully captured by conventional clinical scales. The observed associations between sway-related variables and functional outcomes, particularly under sensorially challenged conditions, suggest that posturography may provide clinically relevant information regarding balance and mobility impairment in individuals with chronic stroke. Furthermore, the results support the integration of posturographic assessment into routine clinical evaluation to guide targeted rehabilitation strategies, such as interventions focusing on sensory integration, mediolateral stability, and dynamic postural control, thereby enhancing individualized treatment planning and optimizing functional outcomes.

### Limitations of the study and areas of future research

Several limitations should be considered when interpreting the findings of this study. The cross-sectional design does not permit causal inference or the determination of longitudinal relationships. The identified posturographic measures should therefore be interpreted as associated factors rather than predictive variables independently associated with functional outcomes within a cross-sectional analytical framework, rather than as definitive determinants of functional recovery. Although the sample size was adequate for the planned analyses, its relatively modest size may limit generalizability, particularly to individuals with more severe stroke-related impairments or those in the acute recovery phase. Furthermore, the study primarily evaluated static and quasi-static posturographic parameters, which may not fully reflect the dynamic balance demands encountered in everyday activities.

Additional factors that may influence postural control and mobility after stroke, including cognitive function, lesion location, stroke severity, and psychological variables such as fear of falling, were not systematically assessed. Fall history was also not prospectively monitored, limiting the ability to compare posturographic characteristics between fallers and non-fallers. Such analyses may provide clinically relevant insight into the potential utility of objective balance measures for identifying individuals at elevated fall risk after stroke. Future longitudinal investigations incorporating prospective fall monitoring, dynamic posturography, real-world mobility assessment, and multimodal evaluations including cognitive and neurophysiological measures may improve understanding of postural control mechanisms and further clarify the clinical value of specific sway parameters, particularly mediolateral instability and sway velocity measures, for rehabilitation planning and fall-risk stratification in chronic stroke populations.

## Conclusion

This study demonstrated that posturographic measures of balance are significantly associated with clinical mobility and functional outcomes in individuals with chronic stroke, with stronger relationships observed under sensory-challenged conditions. Key posturographic variables, particularly those reflecting sway magnitude and mediolateral instability, were identified as significantly independently associated with mobility performance and balance function, whereas stroke duration showed limited influence. These findings underscore the relevance of objective posturographic assessment in capturing clinically meaningful impairments in postural control and their impact on functional performance in this population.

## Data Availability

The datasets generated and/or analyzed during the current study are not publicly available, but will be made available by the corresponding author upon reasonable request.
